# Circulating species of *Leishmania* at microclimate area of Boulemane Province, Morocco: impact of environmental and human factors

**DOI:** 10.1186/s13071-017-2032-9

**Published:** 2017-02-22

**Authors:** Asmae Hmamouch, Mahmoud Mohamed El Alem, Maryam Hakkour, Fatima Amarir, Hassan Daghbach, Khalid Habbari, Hajiba Fellah, Khadija Bekhti, Faiza Sebti

**Affiliations:** 1 National Reference Laboratory of Leishmaniasis, National Institute of Hygiene, Rabat, Morocco; 2 Laboratory of Microbial Biotechnology, Sciences and Techniques Faculty, Sidi Mohammed Ben Abdellah University, Fez, Morocco; 3 Laboratory of Zoology and General Biology, Faculty of Sciences, Mohammed V University, Rabat, Morocco; 4 Institute of Nursing Professions and Health Techniques, Casablanca, Morocco; 5 Delegation of Ministry of health, Provincial Laboratory of Parasitic Diseases, Boulemane, Morocco; 6 Faculty of Sciences and Techniques, Sultan Moulay Slimane University, Beni Mellal, Morocco

**Keywords:** *Leishmania tropica*, *Leishmania major*, Environmental factors, OLSR, Boulemane, Morocco

## Abstract

**Background:**

Cutaneous leishmaniasis (CL) is widely distributed in Morocco where its geographical range and incidence are related to environmental factors. This study aimed to examine the impact of several factors on the distribution of CL in Boulemane Province, which is characterized by several microclimates, and to identify the *Leishmania* species circulating in these areas.

**Methods:**

Ordinary least squares regression (OLSR) analysis was performed to study the impact of poverty, vulnerability, population density, urbanization and bioclimatic factors on the distribution of CL in this province. Molecular characterization of parasites was performed using a previously described PCR-RFLP method targeting the ITS1 of ribosomal DNA of *Leishmania*.

**Results:**

A total of 1009 cases were declared in Boulemane Province between the years 2000 and 2015 with incidences fluctuating over the years (*P* = 0.007). Analyzing geographical maps of the study region identified four unique microclimate areas; sub-humid, semi-arid, arid and Saharan. The geographical distribution and molecular identification of species shows that the Saharan microclimate, characterized by the presence of *Leishmania major* was the most affected (47.78%) followed by semi-arid area where *Leishmania tropica* was identified in three districts. Among several environmental factors included in the study, poverty had the greatest influence on the spatial extension of the disease in this province.

**Conclusions:**

The incidence of CL in Boulemane Province varies between microclimate areas, and environmental factors partly explain this variation. However, the existence of CL in the most affected districts is mainly related to poverty, population movement and human behavior. To our knowledge, this the first study utilizing molecular techniques to confirm *L. tropica* and *L. major* as the causative agents of CL in Boulemane Province. Our findings indicate that the spatial and temporal distribution of CL in Boulemane Province is strongly related to poverty and population movement.

## Background

Leishmaniasis is among the most important emerging vector-borne protozoal diseases in terms of disability adjusted life year (DALY’s). Approximately, 1.5 million cases of cutaneous leishmaniasis (CL) and 500,000 cases of visceral leishmaniasis (VL) are reported each year and approximately 350 million people are at risk [1]. Leishmaniasis is caused by any of several parasites belonging to the genus *Leishmania* (Kinetoplastida: Trypanosomatidae), that are transmitted *via* the bite of phlebotomine sand flies (Diptera: Psychodidae). The disease is represented by three main clinical syndromes: mucocutaneous, visceral and cutaneous leishmaniasis, the latter two being the most common [1]. In Morocco, 2933 cases of CL were declared in 2015 with fluctuations in the number of cases reported over the years [2]. As with other vector-borne diseases, the geographical distribution of cases suggests that CL transmission is sensitive to vector density which is correlated to climatic conditions [3]. Due to its geographical position, Morocco possesses different ecological and climatic conditions [4] which influence the distribution and epidemiology of endemic disease such as leishmaniasis. Based on the bioclimatic map of Morocco, the distribution of sand flies revealed a diversity of species with specific rain-thermal preferences [5].

The objectives of this study are to (i) determine the impact of these environmental conditions on the distribution of CL in this province, (ii) identify the roles of the socio-economic factors on the distribution of CL, and (iii) identify the *Leishmania* species responsible for CL in this province using molecular techniques.

## Methods

### Study area

Boulemane Province (33°3'–33°0'N, 3°59'–3°27'W) belongs to the region of Fes-Boulemane in central Morocco. This province extends over a large surface of 14,395 km^2^ (71% of the region). Administratively, this province is composed of 21 districts (four urban and 17 rural) and is bordered to the north by Sefrou and Taza provinces, to the west by Ifrane Province, to the south by Khenifra, Errachidia and Figuig provinces and to the east by Taourirt Province [6]. Climatically, Boulemane Province contains four bioclimates: (i) a subhumid bioclimate with moderate winters and precipitation varying from 700 to 800 mm with a mean temperature of 13.9 °C; (ii) a semi-arid bioclimate characterized by a cold winter (rainfall is about 450 mm per year and mean temperature of 15.6 °C) and hot summers; (iii) an arid bioclimate with very cold winters (rainfall is about 130 mm per year and an average temperature of 16.7 °C); and (iv) a Saharan bioclimate with rainfall of approximately 74 mm per year and a mean temperature of 17.6 °C [7]. Geographically, this province is divided into two areas: a mountainous area that includes the mountain of Tichoukt with a maximum elevation of 2,796 m, and is crossed by the major rivers of M’daz, Guigou and Maasar and a pre-Saharan area that includes plains, valleys and the high plateaus of Moulouya where there is irrigated agricultural land. Olives and fruit are the main agricultural commodities and are of significant economic importance to this province. Boulemane Province is famous for its deposits of ghassoul located near Ksabi District, which extends over 25,000 ha [6]. This province had a total of 197,596 inhabitants in 2014 [8].

### Epidemiological data and statistical analyses

The epidemiological study was performed using 1009 cases of CL reported between 2000 and 2015, clinically diagnosed in the health center of Boulemane Province. Data related to human cases of leishmaniasis were provided by the Directorate of Epidemiology and Disease Control [2]. Data on the poverty rate, vulnerability rate, population density and the two degrees of urbanization (rural/urban) were established for each district of Boulemane Province. These data were obtained from the High Commission for Planning [9]. Data concerning the bioclimatic areas in this province were provided by the agency of Sebou Hydraulic basin and from the Ministry of Housing and Urban Planning in Morocco [10].

Statistical analysis was performed using the IBM SPSS Statistical software version 24.0. The ordinary least squares regression model was applied to assess the impact of several factors: poverty rate, vulnerability rate, population density, urbanization and bioclimatic area on the distribution of leishmaniasis in Boulemane Province. For all statistical methods, a cut-off of *P* = 0.05 was used.

### Molecular methods

In outbreaks of leishmaniasis, the consultants of the World Health Organization recommend that health professionals make a clinical diagnosis and microscopically confirm the first cases of leishmaniasis declared in suspected outbreak. It is recommended that later cases receive treatment based on a clinical diagnosis in the absence of a microscopic diagnosis [11]. Consequently, our laboratory received stained slides for approximately 47% of leishmaniasis cases declared (59 cases) during 2015 in Boulemane Province (28 slides in total). These slides were subjected to DNA extraction using a Qiagen DNA minikit, according to the manufacturer’s instructions (Qiagen, Hilden, Germany) [12]. The internal transcribed spacer (ITS1) region was amplified using primers LITSR: 5′-TGATACCACTTATCGCACTT-3′ and L5.8S: 5′-CTGGATCATTTTCCGATG-3′ [13]. Amplification of the target genes was carried out in a total volume of 25 μl containing 20 pmol/μl of each primer (Eurofins MWG Operon Anzingerstrabe 7a D-85560, Ebersberg, Germany), 5 unit/μl of *Taq*polymerase (Promega, Adison, USA), 10 Mm of each DNTPs and 25 mM of MgCl_2_, and5μl of DNA. The cycling program was initiated with an initial denaturation of 5 min at 95 °C, followed by 32 cycles of 94 °C for 30 s, 60 °C for 45 s, 72 °C for 60 s, then a final extension step at 72 °C for 5 min. The ITS1amplicon was subjected to restriction fragment length polymorphism (RFLP) analysis using the endonuclease HaeIII as previously described [13]. Reference strains of *Leishmania infantum* (MHOM/MA/1998/LVTA),*Leishmania tropica* (MHOM/MA/2010/LCTIOK-4) and *Leishmania major* (MHOM/MA/2009/LCER19-09) were included in all analyses for comparison.

## Results

### Epidemiology of leishmaniasis in Boulemane Province

#### Temporal distribution of CL cases in Boulemane Province from 2000 to 2015

**Fig. 1:**
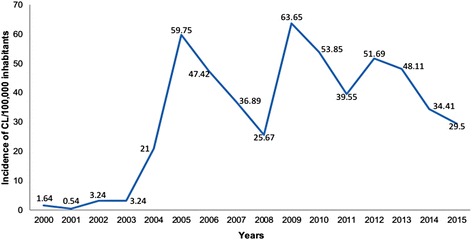
Incidence rate of CL/100,000 inhabitants in Boulemane Province between 2000 and 2015

In Boulemane Province the incidence of CL was low between the years 2000 and 2004 with an average six cases/100,000 inhabitants. In 2005, an increase of cases was observed with 59.75 cases/100,000 inhabitants. Thereafter, the incidence of leishmaniasis decreased to 25.67 cases/100,000 inhabitants in 2008. An increase in cases was noted in 2009 with 63.65 cases/100,000 inhabitants followed by a decrease in the last 2 years of the study (Fig. 1). The variation of cases between the years was significant according to the OLSR analysis (*P*-value = 0.007) (Table 1).

**Table 1:** OLSRof human leishmaniasis cases, and environmental factors in Boulemane Province

Factor	Variable	Coefficient	*t*-statistic	*P*-value
Year	Intercept	-11,370.830	-3.175	0.007
	Year	5.696	3.192	0.007
		Adjusted *R* ^2^ = 0.380	*F*_(1,14)_ = 10.194	*F*_(1,14)_ = 0.007
Microclimate area	Intercept	3,640.333	3.530	0.176
	Microclimate area	-159.000	-3.240	0.191
		Adjusted *R* ^2^ = 0.826	*F*_(1,1)_ = 10.497	*F*_(1,1)_ = 0.191
Poverty	Intercept	-631.73	-0.47	0.654
	Poverty rate	199.99	2.28	0.04
		Adjusted *R* ^2^ = 0.23	*F*_(1,13)_ = 5.19	*F*_(1,13)_ = 0.04
Density of population according to districts	Intercept	60.438	1.519	0.157
	Districts	-4.380	-1.403	0.188
	Population	0.000	-0.298	0.772
		Adjusted *R* ^2^ = 0.238	*F*_(2,11)_ = 3.028	*F*_(2,11)_ = 0.090
Urbanization and vulnerability	Intercept	234.167	2.057	0.055
	Urbanization	25.221	0.545	0.593
	Vulnerability rate	-9.344	-1.855	0.081
		Adjusted *R* ^2^ = 0.30	*F*_(2,18)_ = 1.205	*F*_(2,18)_ = 0.338

#### Geographical distribution of leishmaniasis in Boulemane Province from 2000 to 2015

**Fig. 2:**
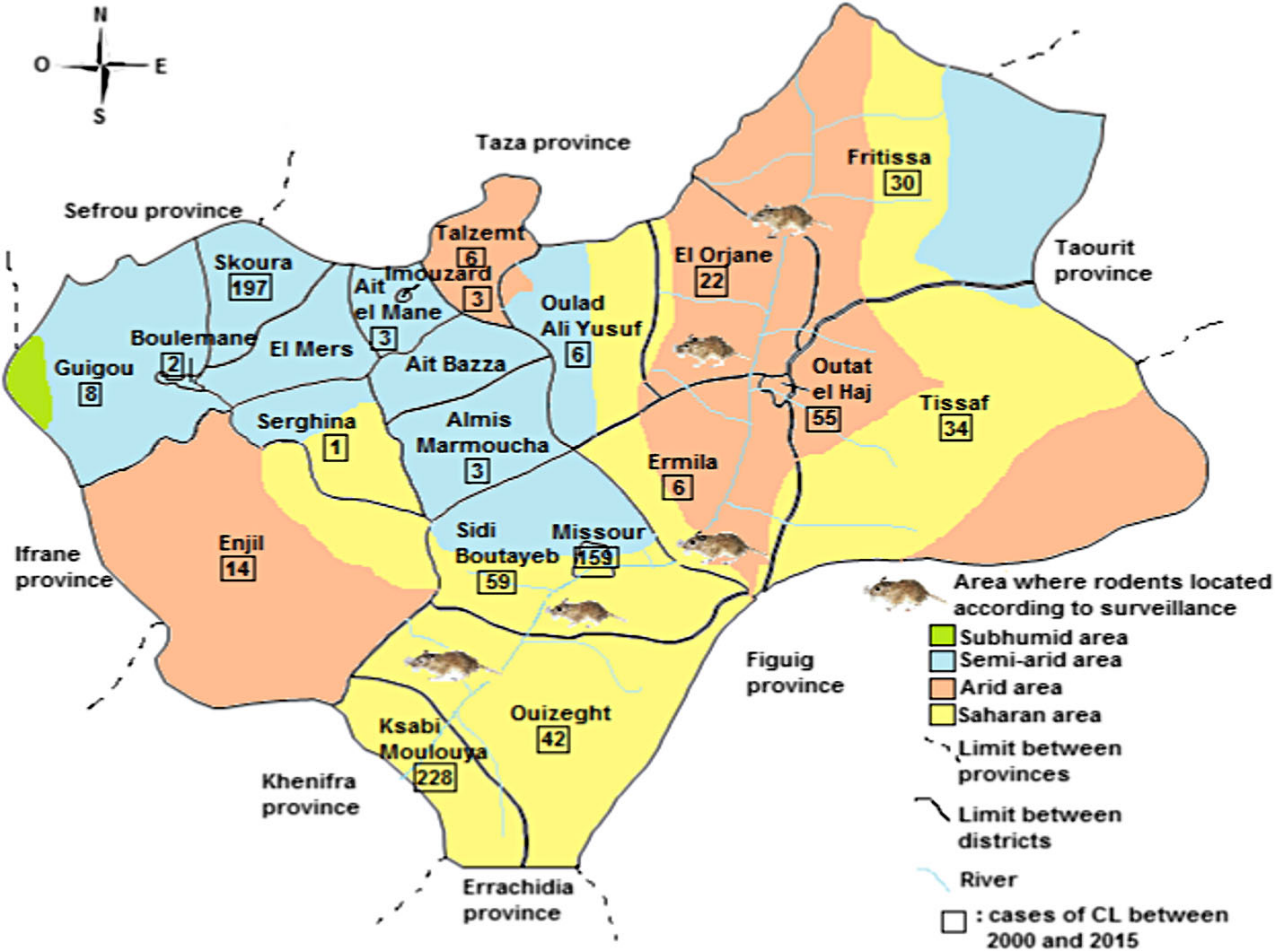
Distribution of CL according to microclimate area from 2000 to 2015 in Boulemane Province

In Boulemane Province CL was reported in each of the four bioclimatic areas between 2000 and 2015 (Fig. 2). The Saharan microclimate was the most affected with 47.78% (432 cases) of all cases reported, 53% occurring in the rural district of Ksabi Moulouya. In the semi-arid area, 358 cases of CL (39.6%) were distributed in 11 districts with more than half of all cases (55%) in Skoura districts. In arid areas, 114 cases (12.61%) were spread over six districts. The OLSR analysis (Table 1) revealed that the local microclimate has little effect on the number of cases of CL in this province.

**Fig. 3:**
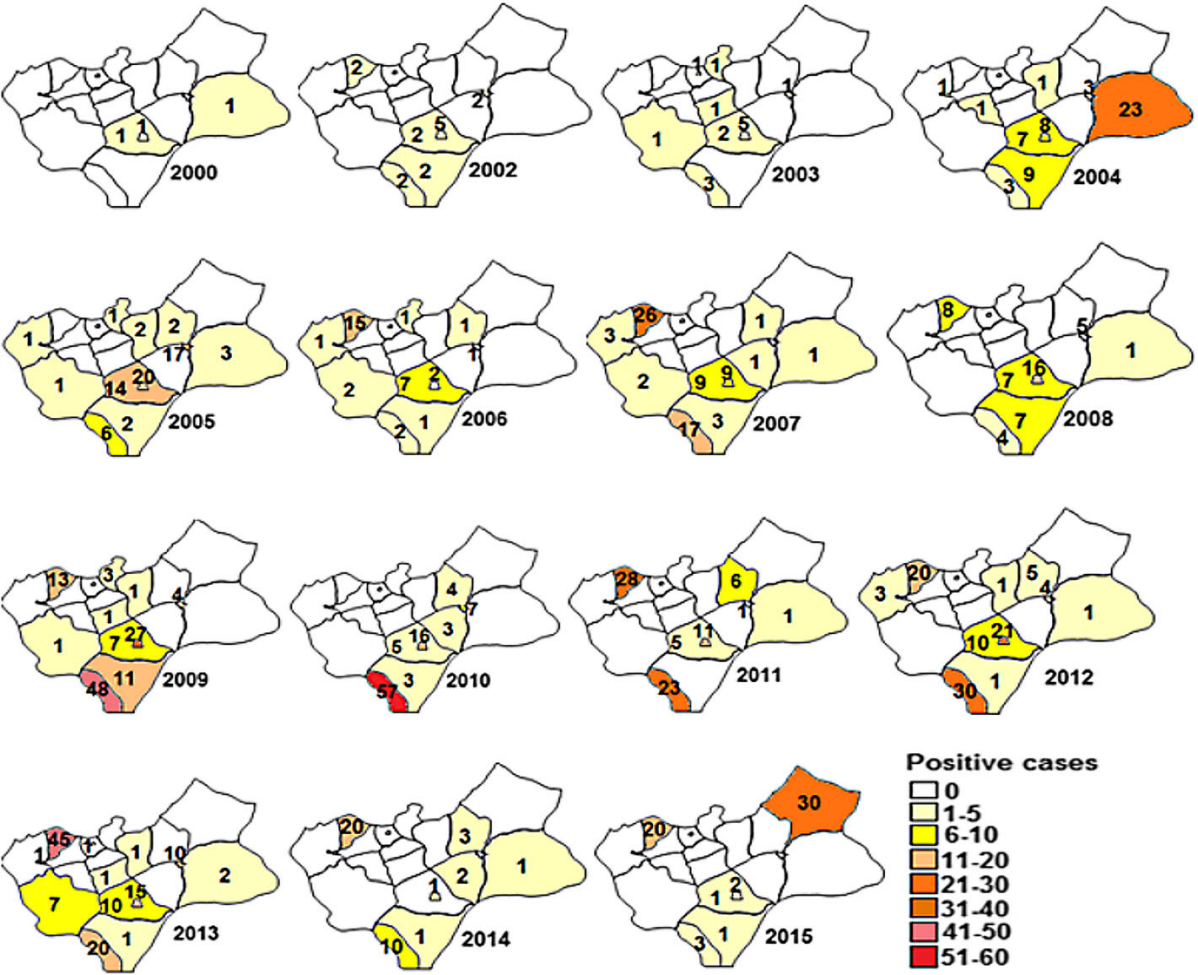
Geographical distribution of CL cases in Boulemane Province, between 2000 and 2015

Over the years, the spatial distribution of leishmaniasis (Fig. 3) showed that the disease was detected in 19 of the 21 districts in this province between the years 2000 and 2015. Between 2000 and 2004, the disease was limited to ten districts with an average of 4–5 cases/year. From 2005 to 2012 the disease was detected in 18 districts, though with most cases occurring in the districts of Ksabi, Skoura and Missour. By 2014, the incidence of leishmaniasis was relatively low in most district sexcept for Skoura district. The incidence of CL in Fritissa district was considered epidemic in 2015.

#### Impact of environmental factors on the distribution of leishmaniasis in Boulemane Province

**Fig. 4:**
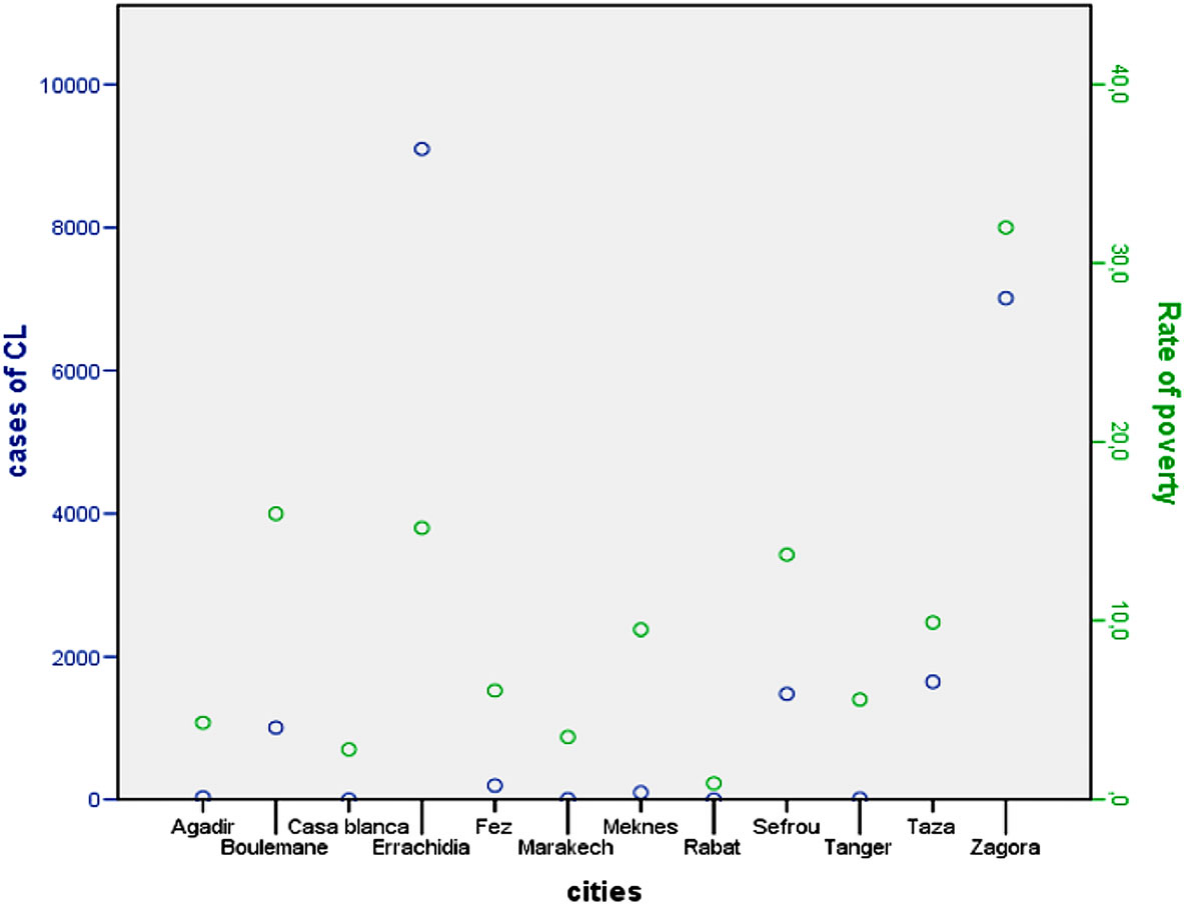
Plot of distribution of leishmaniasis according to rate of poverty in Morocco

**Fig. 5:**
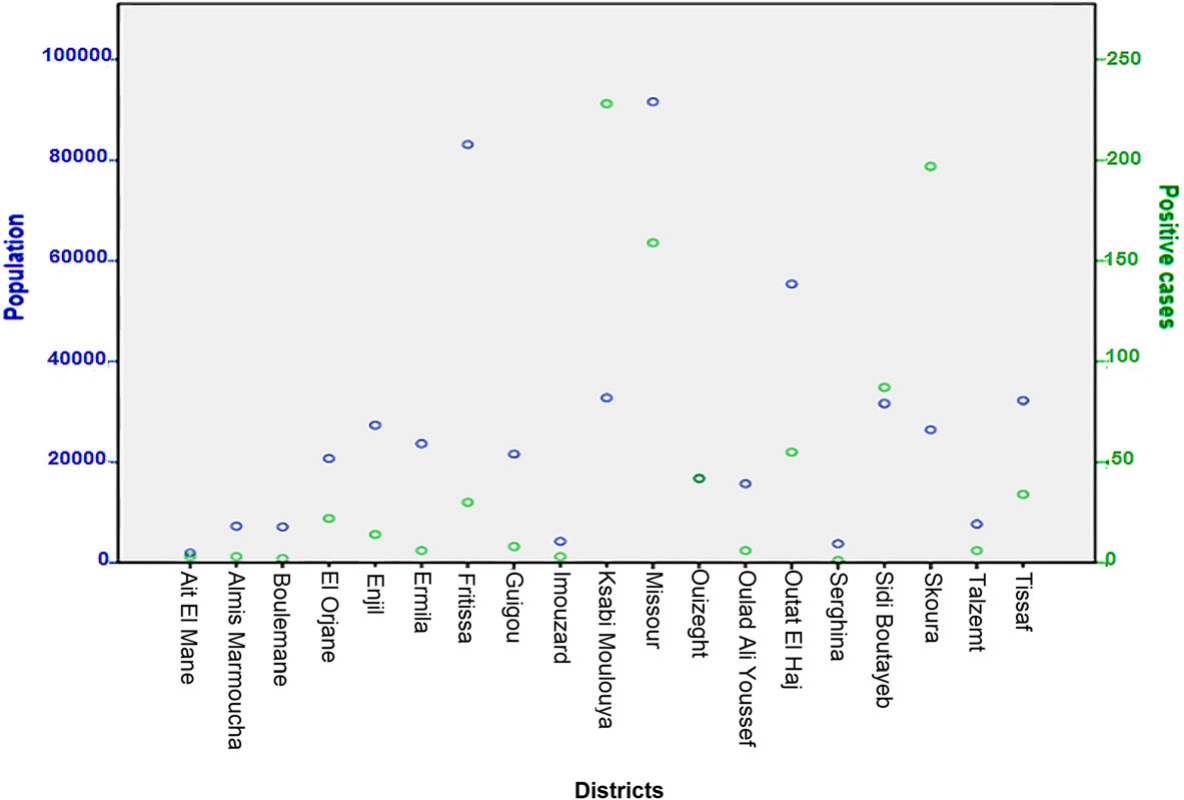
Plot of distribution of CL according to population in Boulemane Province

**Fig. 6:**
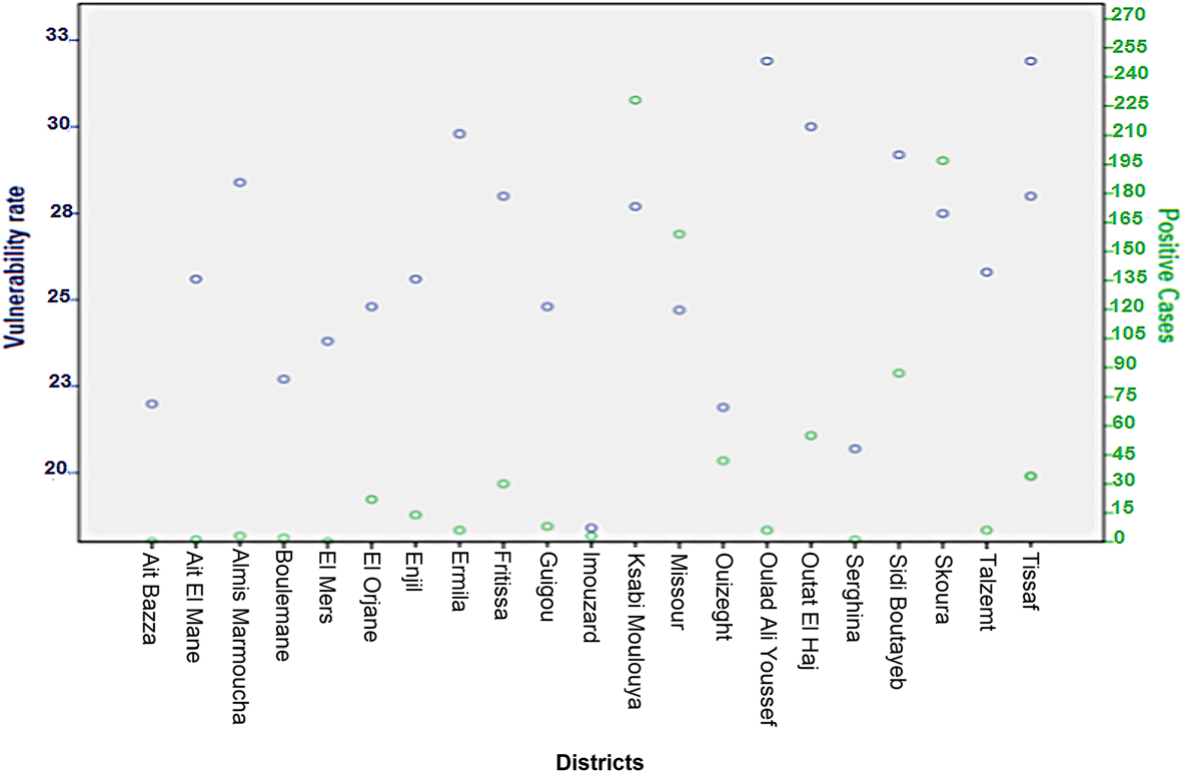
Plot of distribution of leishmaniasis according to vulnerability rate in Boulemane Province

OLSR showed that poverty had a significant effect on the incidence of leishmaniasis (Fig. 4, Table 1). However, the distribution of leishmaniasis in Boulemane Province was not correlated to population density (Fig. 5, Table 1), nor was it affected by the vulnerability rate (Fig. 6, Table 1) or urbanization (Table 1).

#### Distribution of CL in Boulemane Province according to age, sex and clinical features

**Fig. 7:**
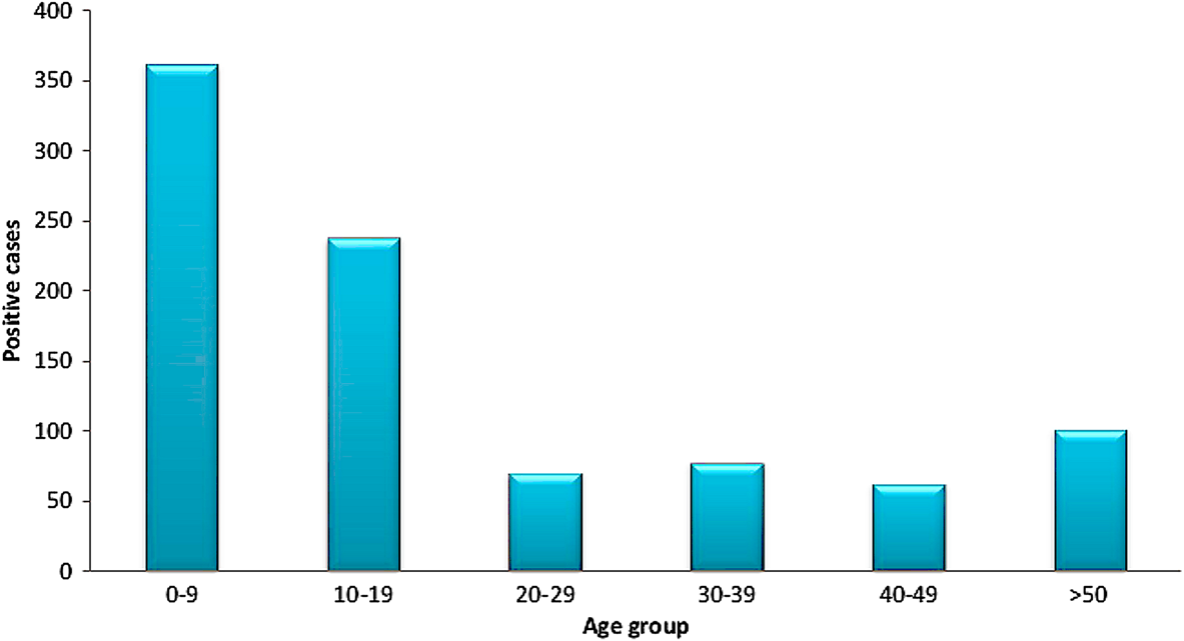
Distribution of CL cases according to age in Boulemane Province (2000–2015)

**Fig. 8:**
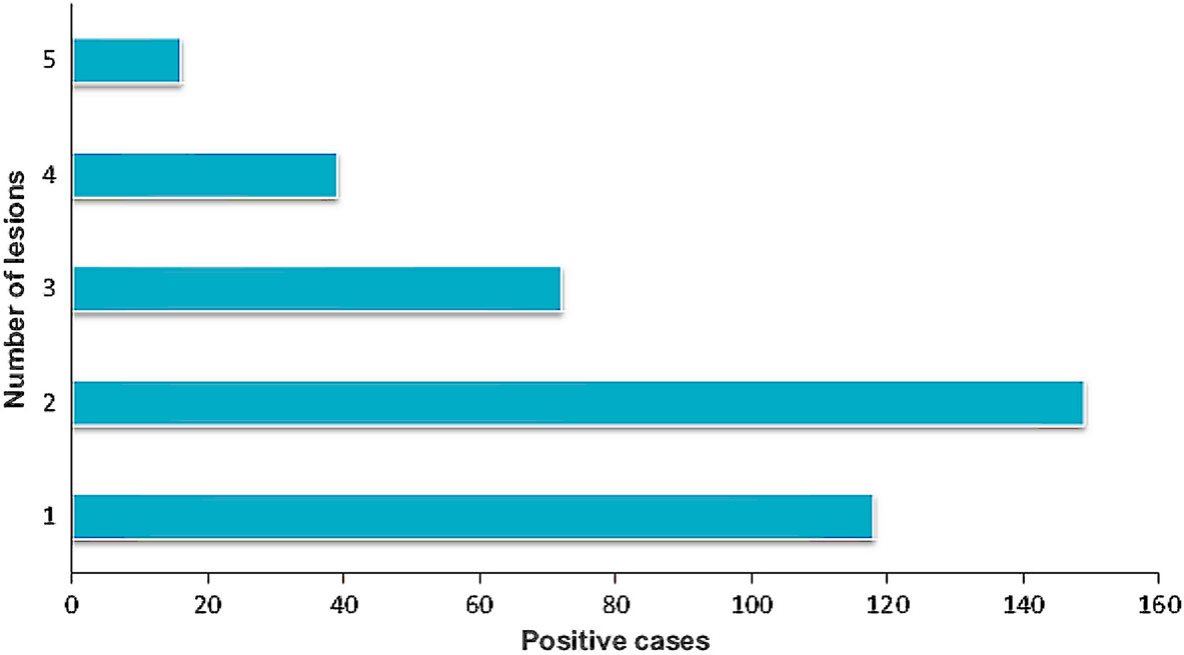
Distribution of CL cases according to number of lesions in Boulemane Province (2000–2015)

**Fig. 9:**
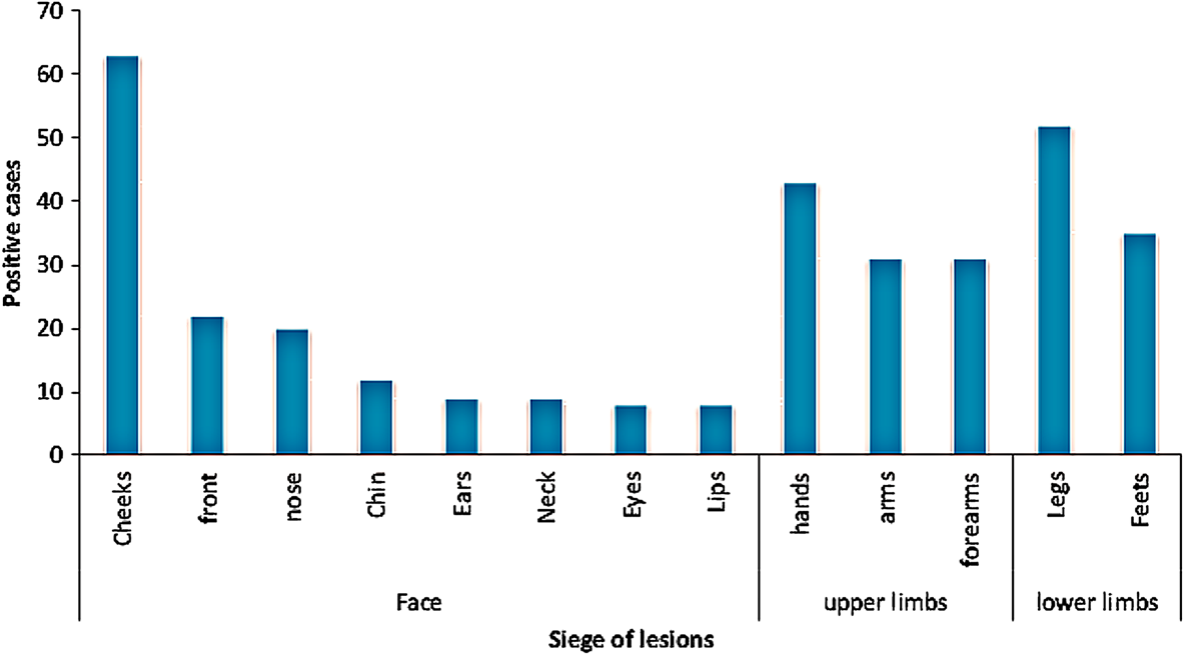
Distribution of CL cases according to siege of lesions in Boulemane Province (2000–2015)

**Fig. 10:**
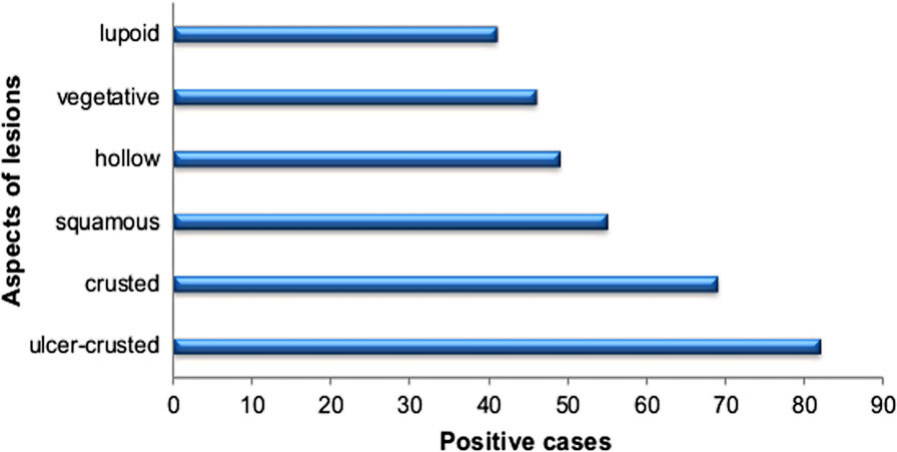
Distribution of CL cases according to aspect of lesions in Boulemane Province (2000–2015)

The average age of *Leishmania* infected individuals was 21 years with a minimum of 6 months and a maximum of 95 years old (Fig. 7). The age group most represented was that of 0–9 years old with 362 cases. The age group of 10–19 years was in the second position with 238 cases. The age group 20–49 years was affected with an average of 69 cases. In this province 101 patients were older than 50 year-old. The distribution of cases by sex was uniform with 51% female and 49% are male. The number of lesions ranged from 1 to 5 lesions (Fig. 8). Most patients had two lesions (37.8%), followed by single lesions (30%). Multiple lesions were observed in 29% of patients. Most of lesions were observed on the face (44%), followed by the upper limbs (30.6%). In the lower limbs 25.4% of lesions were located (Fig. 9). The face and cheeks were the most frequent site of exposure (42%) followed by the forehead with 14.5%, and nose with 13%. Other lesions mentioned on the face were on the chin, ears, eyes and lips. On the upper limbs, the hands were most commonly affected with 41% followed by the arm and forearm with 29.5% each. Finally, the legs were the preferred bite site of sandflies (60%) on the lower limbs. The clinical aspect of lesions was examined in 342 patients (Fig. 10) where six aspects were identified. Ulcerative and crusted lesions predominated with 24 and 20%, respectively. The scaly lesions were observed in 55 cases (16%) followed by hollow, vegetative and lupoid lesions with an average of 13%.

### Molecular study

**Fig. 11:**
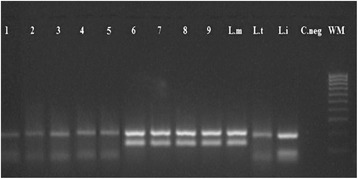
Application of RFLP analysis of the ITS1-PCR analysis on positive amastigotes slides from Boulemane province. Lanes 1–9: samples from slides. *Abbreviations*; Lt, *L. tropica*; Li, *L. infantum*; Lm, *L. major*; C.neg, negative control; WM, weight maker 100 bp

Of the 59 cases of CL declared in 2015, 28 were analyzed microscopically and were positive. All of them were positive after ITS1PCR-RFLP. *Leishmania major* was identified in five samples belonging to three different districts (Ksabi Moulouya, Sidi Boutayeb and Ouizeght) situated in the Saharan microclimate area. Concerning *L.tropica*, it was identified in three other districts (Missour, Fritissa and Skoura) owing to their Saharan and semi-arid microclimates (Table 2, Fig. 11).

**Table 2:** Results of molecular study of CL from the most affected district in Boulemane Province in 2015

Province	District	Locality	Bioclimatic area	Urban/Rural	Result of ITS1	Result of RFLP	Number of slides
Boulemane	Ksabi Moulouya	Akhachab	Saharan	Rural	Positive	*L. major*	1
Boulemane	Ksabi Moulouya	Issararen	Saharan	Rural	Positive	*L. major*	1
Boulemane	Sidi Boutayeb	Bsabis	Saharan	Urban	Positive	*L. major*	1
Boulemane	Ouizeght	Brija	Saharan	Rural	Positive	*L. major*	2
Boulemane	Missour	Hay el qods	Saharan	Urban	Positive	*L. tropica*	1
Boulemane	Missour	Pam	Saharan	Urban	Positive	*L. tropica*	1
Boulemane	Fritissa	Oulad rzagh	Semi-arid	Rural	Positive	*L. tropica*	8
Boulemane	Fritissa	oulad gharsalh	Semi-arid	Rural	Positive	*L. tropica*	6
Boulemane	Fritissa	Oulad lahcen	Semi-arid	Rural	Positive	*L. tropica*	1
Boulemane	Skoura	Bouassem	Semi-arid	Rural	Positive	*L. tropica*	2
Boulemane	Skoura	Taghrout	Semi-arid	Rural	Positive	*L. tropica*	2
Boulemane	Skoura	Skoura	Semi-arid	Rural	Positive	*L. tropica*	2

## Discussion

Leishmaniasis is a dynamic disease and the circumstances of transmission are continually changing in relation to environmental, demographic, socio-economic and human behavioral factors [14]. The only study performed in Boulemane Province identified *L. tropica* in seven samples without specifying their origin [15]. Our study aimed to determine the impact of several factors on the distribution of leishmaniasis by molecular epidemiological methods to identify the circulating species according to microclimate area affected in 2015. The results of our study suggest that the microclimate does not affect the number of cases of leishmaniasis (Table 2). However, the microclimate does appear to affect the distribution of *Leishmania* species [5]. *Leishmania tropica* was identified in Skoura, Missour and Fritissa. In the first district, this result could be explained by its semi-arid microclimate and its mountainous areas in which *Phlebotomus sergenti* was the most commonly collected sandfly species in a previous report (60.4%) followed by *Phlebotomus perniciosus* and *P.longicuspis* (18.7 and 11.2% respectively) [16]. The temporal evolution of CL cases due to *L. tropica* did not decrease over the years in Skoura district despite actions of control implemented in 2012, according to the delegation of health in Boulemane Province (eradication of sandflies and the early management of cases). In Morocco, the transmission cycle of *L. tropica* is still not well elucidated since it was isolated from dogs in Azilal and Essaouira provinces [17, 18]. Therefore, control measures should consider the zoonotic transmission cycle of this species. The spatial distribution of leishmaniasis due to *L. tropica* has extended into Skoura. This could be due to the population movement from neighboring foci such as Sefrou Province and Taza [19, 20]. This displacement of people is related to socio-economic factors namely the presence of a weekly market and olive crushing factories which constitute a meeting place for the local populace, including those from neighboring provinces. These meetings provide good conditions for transmission of the disease. In Missour district owing to semi-arid and Saharan areas, the two cases declared in 2015 were identified as *L. tropica* on the Saharan side after the elimination of *L. major* following the successful implementation of rodent control strategies. The presence of *L. tropica* could be due to the presence of its vector *P. sergenti* at lower densities and dominated previously by *P. papatasi* in this microclimate. This same result was found in Errachidia Province where the same action of control was implemented against rodents [21]. In 2015, an epidemic of CL was declared in Fritissa District (previously an unaffected area) belonging to several microclimate areas (semi-arid, arid and Saharan). In the context of public health surveillance, we have identified *L. tropica* in the three localities on the semi-arid side of this district. These localities are characterized by unfavorable conditions of hygiene which promote the presence of reservoir hosts and vectors and thus increase the risk of CL transmission.

*Leishmania major* was identified in the Saharan area in three affected districts in 2015, namely Ksabi Moulouya, Ouizeght and Sidi Boutayeb. These districts are situated at an altitude of 400 to 600 m where *Phlebotomus papatasi* predominates [5]. According to other studies, *L. major* is in circulation in Saharan areas [14, 22, 23]*.* The presence of *L. major* in all these districts could be explained by the geographical features of these districts characterized by the presence of River Moulouya, sandy soil and desert plants that constitute suitable environmental conditions for the spread of the disease since it facilitates the construction of rodent burrows; rodents being an important reservoir host [24]. Another factor which could increase the risk of leishmaniasis in Ksabi Moulouya District is the presence of a stone-pit (25,000 ha) which is an artificial environment that facilitates the breeding of sandflies. The temporal evolution of CL due to *L. major* in this province revealed fluctuations in cases over the years with an epidemic cycle of 4 to 5 years. This could be due to the application and looseness of measures of control focused mainly on rodents.

Cases of leishmaniasis due to *L. major* and *L.tropica* are related to poverty (*P*-value = 0.04) (Fig. 4). Other studies also support the relationship between the increase risk of CL and the poverty [25, 26]. Moreover, our results highlight that the distribution of leishmaniasis was not influenced by urbanization since CL affects both urban and rural areas. In relation tourban areas, 159 cases were recorded in Missour District which could be explained by its position as the administrative and commercial center of the province. Consequently, this district constitutes an area to which individuals from other neighboring affected foci, regularly travel to and meet. Also, this district is characterized by its high population density which promotes the installation of new settlements in the poor suburbs of this district, leading to inadequate sanitation and housing thus creating opportunities for the transmission of *Leishmania*. In rural areas, the increase of CL cases could be linked to human behavior through human-animal coexistence and the accumulation of livestock waste near habitations [27]. These factors represent favorable conditions for the spread of reservoir hosts and vectors [28, 29].

On the other hand, the OLSR analysis showed that the population density does not affect the incidence of the disease in this province (Fig. 5). Indeed, districts such as Enjil and Ermila with high population densities recorded a small number of cases (six and 14 cases, respectively) from 2000 to 2015. These districts are far from the leishmaniasis foci and the distance between their localities is wide which explains the stability of the population and therefore the low possibility of transmission.

During the study period two districts (Ait Bazza and El Mers) were not affected by the disease, but since these districts belong to the same biotope of other CL foci in this province such as Skoura District, the possibility of transmission still exists, should other factors (especially human behavior) allow it.

According to the clinical study of CL in Boulemane Province, the distribution of CL by age showed that all age groups were affected by the disease with the dominance of children from 0 to 9 years. This dominance could be explained by the relative immaturity of the cellular immunity of children and consequently the inability to fight the *Leishmania* infection [30]. Also, this could be due to the habits of children who often play near breeding sites [31]. Concerning the clinical characterization, the results obtained were comparable to previous studies [15, 18]. We observed the dominance of multiple lesions on the face and upper limbs. The multiplicity of lesions and their location on the limbs represents the classical form of CL due to *L.major* [1, 18]. When a sandfly is disturbed during its blood meal, it can take its meal at multiple sites on the same host which could explain the multiplicity of lesions. This behavior is observed mainly in *P.papatasi* [32]. In this province, a diversity of clinical signs was observed generally indicative of CL due to *L. major*, namely ulcer-crusted and vegetative lesions [15, 18]. Lupoid lesions are also observed in this province which is a characteristic of CL due to *L.tropica* [33].

## Conclusions

It appears that the increase of transmission of CL in Boulemane Province is related to several environmental factors such as poverty and population movement. The strength of this molecular epidemiological study lies in the molecular identification of the circulating species at each microclimate which can guide the control and surveillance strategies implemented to manage CL in this province.

## Abbreviations

CL: Cutaneous leishmaniasis
NRLL: National Reference Laboratory of Leishmaniasis
OLSR: Ordinary least squares regression
